# Spatial-attention ConvMixer architecture for classification and detection of gastrointestinal diseases using the Kvasir dataset

**DOI:** 10.1007/s13755-024-00290-x

**Published:** 2024-04-28

**Authors:** Ayşe Ayyüce Demirbaş, Hüseyin Üzen, Hüseyin Fırat

**Affiliations:** 1 Ankara, Turkey; 2 Department of Computer Engineering, Faculty of Engineering, Bingol University, Bingol, Turkey; 3 Department of Computer Engineering, Faculty of Engineering, Dicle University, Diyarbakır, Turkey

**Keywords:** Gastrointestinal endoscopy images, ConvMixer, Spatial attention mechanism, Kvasir dataset

## Abstract

Gastrointestinal (GI) disorders, encompassing conditions like cancer and Crohn’s disease, pose a significant threat to public health. Endoscopic examinations have become crucial for diagnosing and treating these disorders efficiently. However, the subjective nature of manual evaluations by gastroenterologists can lead to potential errors in disease classification. In addition, the difficulty of diagnosing diseased tissues in GI and the high similarity between classes made the subject a difficult area. Automated classification systems that use artificial intelligence to solve these problems have gained traction. Automatic detection of diseases in medical images greatly benefits in the diagnosis of diseases and reduces the time of disease detection. In this study, we suggested a new architecture to enable research on computer-assisted diagnosis and automated disease detection in GI diseases. This architecture, called Spatial-Attention ConvMixer (SAC), further developed the patch extraction technique used as the basis of the ConvMixer architecture with a spatial attention mechanism (SAM). The SAM enables the network to concentrate selectively on the most informative areas, assigning importance to each spatial location within the feature maps. We employ the Kvasir dataset to assess the accuracy of classifying GI illnesses using the SAC architecture. We compare our architecture’s results with Vanilla ViT, Swin Transformer, ConvMixer, MLPMixer, ResNet50, and SqueezeNet models. Our SAC method gets 93.37% accuracy, while the other architectures get respectively 79.52%, 74.52%, 92.48%, 63.04%, 87.44%, and 85.59%. The proposed spatial attention block improves the accuracy of the ConvMixer architecture on the Kvasir, outperforming the state-of-the-art methods with an accuracy rate of 93.37%.

## Introduction

Gastrointestinal (GI) disorders are prevalent in the human digestive system and pose a significant threat to public health. These disorders include cancer, bleeding, ulcer polyps, Crohn’s disease, and they are a serious concern in today’s world [1]. Esophageal, stomach and colorectal cancer are among the most commonly diagnosed and lethal types of cancer worldwide [2]. To diagnose and treat these disorders, endoscopic examinations have become a crucial diagnostic tool. Endoscopy, an efficient medical imaging technique, excels in identifying irregularities within the GI tract [3]. In addition to aiding in disease diagnosis, endoscopy also helps to confirm findings and treat certain abnormalities [4]. Endoscopy is a minimally invasive operation that uses a flexible, thin, and elongated tube called an endoscope to visualize the internal organs of the patient. The endoscope is equipped with a camera and a light source to transmit images of the organs to a monitor, allowing for accurate diagnosis and treatment planning. Depending on the specific aim of the operation, the equipment used and the internal structures being examined, there are diverse types of endoscopy. The endoscope can be inserted through the mouth and throat or via a small incision in the skin [5, 6].

The human GI system encounters a range of unusual mucosal symptoms, spanning from minor issues to extremely severe illnesses. Thus, accurate and timely diagnosis is fairly important with efficient treatment and reducing mortality rates. Endoscopic evaluations play a very important role in identifying abnormalities in the human GI tract. Thanks to these evaluations, the severity and type of clinical features of GI disease are known and treatment methods are determined according to appropriate diagnoses. However, endoscopic examination of the disease and classification of different symptoms are done by gastroenterologists. One of the important responsibilities of a gastroenterologist (GE) involves studying and analyzing images and videos of the GI system [7]. However, endoscopic examination of the disease and classification of different symptoms may differ from one GE to another, depending on the symptoms as a result of the analysis of GI tract images [8, 9]. These differences may cause errors in some cases, particularly regarding controversial directions of diagnostic videos and images obtained from endoscopic examinations. Such errors can lead to misdiagnosis of the disease. In this direction, various studies have been conducted on automatic classification systems for diagnosing GI tract-related diseases from endoscopic images [10]. Automated classification of diseases presents a promising resolution by ensuring GEs have dependable and useful support in recognizing GI endoscopic images. This, in turn, reduces the occurrence of misdiagnoses and conserves valuable time for gastroenterologists. Consequently, the automated classification of GI illnesses remains a substantial area of research aimed at enhancing the precision of disease detection [11].

The utilization of artificial intelligence (AI)-based systems for the early detection of abnormalities in medical images has attracted great interest in recent years [12–14]. These systems typically employ techniques for feature selection, feature extraction, and classification of medical images, such as wavelet transform features [15], color features [16], texture features [17], point features [18], HOG features [19], and others, to extract relevant image features. Following feature selection and extraction, deep learning (DL) or machine learning (ML) based classifiers can be utilized to classify endoscopic images. Unlike traditional ML-based classifiers used for feature extraction, features are extracted automatically with DL. With this feature, DL and particularly convolutional neural networks (CNNs) offer very successful solutions for more accurate classification for medical imaging [20, 21]. Hence, techniques built upon CNNs have become the preferred and commonly utilized methods in the field of medical image examination.

In this study, a customized Spatial-Attention ConvMixer (SAC) model is proposed for the classification of GI diseases from endoscopy images. The proposed SAC model presents a new DL model by combining the spatial attention mechanism and ConvMixer architecture. In order to test the effectiveness of the proposed SAC model, extensive analyses were performed on the Kvasir dataset. The dataset contains high-resolution endoscopy images of various GI diseases and normal tissues. However, the sample imbalance in this dataset is a challenge that can directly affect the performance of deep learning methods. To overcome this challenge, our study adopts various data augmentation strategies to improve the performance of DL methods and strengthen their generalization capabilities. The principal contributions of this study can be outlined as follows:

1. In this study, a hybrid model is proposed that combines the state-of-the-art ConvMixer architecture and the spatial attention mechanism. This proposed hybrid model is named as Spatial-Attention ConvMixer (SAC) model.
2. In the proposed SAC model, the spatial attention mechanism before the depthwise convolution in ConvMixer, which provides the network to selectively focus on the most informative regions in the input images. This mechanism improves the ConvMixer’s performance in capturing the relevant features of GI diseases, thus enhancing its ability to diagnose and classify the diseases accurately. On the other hand, the ConvMixer architecture processes these regions to obtain strong features. In the final layer of the model, these features are used to produce the classification prediction.
3. To the best of our knowledge, the SAC model is the first attempt to implement a spatial attention mechanism to the ConvMixer architecture for medical image analysis, specifically in the context of GI diseases. Our SAC model acquires state-of-the-art performance on the Kvasir dataset, with an accuracy rate of 93.37%, outperforming the existing methods by a significant margin. Therefore, our SAC model offer a promising solution for the automatic diagnosis and classification of GI diseases, with potential applications in other medical image analysis tasks.

The structure of this paper is organized in the subsequent manner. Related works section provides an overarching review of recent research in our field. Materials and methods section delves into the dataset employed, the SAC architecture, and the associated theoretical underpinnings. Section 4 will address the conducted experiments and their outcomes. Ultimately, Sect. 5 offers a comprehensive summary of our study.

## Related works

In recent years, there have been numerous studies proposing DL approaches for medical image analysis in the field of gastroenterology. One of the common objectives of these studies is to automate the diagnosis of GI diseases using endoscopic images, which can increase diagnostic accuracy and decrease the workload of endoscopists. Recent studies of GI tract abnormalities with endoscopic images have shown that manual evaluation of multiple endoscopic images is laborious and requires expertise. In this direction, efficient intelligent DL, particularly CNN-based architectures have been developed to assist gastroenterologists in their tasks. Thanks to these methods, correct treatment recommendations are shown by automatically extracting the image features through convolutions, processing and analyzing the image data. Also, the use of CNN-based methods showed better classification performance in feature extraction, making them cutting-edge for deep learning applications. The efficient use of CNN has developed tasks related to image classification and recognition. Some of the studies using CNN in the literature are given below.

Poudel et al. [22] developed a powerful architecture for endoscopic image classification using a DL approach. The proposed architecture incorporates an efficient dilation in CNNs to preserve spatial details and prevent loss of information, which can result in the misclassification of similar-looking images and polyps. In addition, the paper introduces a regularization method called DropBlock to address the problem of overfitting and deal with artifacts and noise. The experiments demonstrate that the proposed architecture outperforms traditional architectures and achieves an F1-score of 88% for Kvasir dataset and 93% for Colorectal dataset, indicating its potential to increase the accuracy of endoscopic colon disease classification. Amin et al. [23] developed an automated method for detecting different types of stomach infections using a new deep semantic segmentation method. The method employs deeplabv3 as the backbone of the ResNet-50 method and correctly implements pixel-wise classification of the lesion regions, which are challenging due to their size, irregular shape and low contrast. The method reached up to 90% prediction values, demonstrating its effectiveness in accurately classifying stomach infections and highlighting the potential of uncertainty-aware deep CNNs for improving the diagnostic accuracy of GI infections. Srivastava et al. [24] developed a focal modulation network (FocalConvNet) combined with light convolutional layers, for the classification of small intestinal lumen findings and anatomical landmarks. Following the experimental studies on Kvasir-Capsule, they obtained 63.73% classification accuracy. Liu et al. [25] enhancements were made to a medical image segmentation technique that involves multi-scale feature memory, hybrid attention-driven residual atrous convolution and multi-receptive field fusion module. By applying the technique to the Kvasir dataset to assess its classification accuracy, they achieved an F1-score of 76.65% for polyp segmentation. Lonseko et al. [26] proposed a deep CNN for the classification of GI diseases on endoscopic images using an efficient spatial attention mechanism. In the experimental analyses performed on the Kvasir dataset, which consists of a multi-class structure, 93.19% classification accuracy and 92.8% F1-score values were found. Du et al. [27] developed a semi-supervised effective comparative learning classification architecture for esophageal disease. With this architecture, 92.57% accuracy was achieved in experimental studies.

Along with the development of CNN-based methods, there are studies in the literature using transfer learning with pre-trained CNN architectures. Ahmed et al. [28] developed a architecture for medical image classification using denoising CNNs (DnCNNs) and transfer learning with pre-trained CNNs. The architecture employs AlexNet, a well-known pre-trained CNN, as the classification model and DnCNNs as the pre-processing tool for the Kvasir dataset, which includes endoscopic images. The outcomes reveal that the DnCNNs attained a classification accuracy of 90.17%, surpassing several comparable cutting-edge techniques. Kahsaygebreslassie et al. [29] improved a DL approach for identifying and classifying different GI tract diseases in endoscopic images. The authors have fine-tuned two popular CNN methods, DenseNet121 and ResNet50, on the publicly available Kvasir dataset that contains GI endoscopic images belonging to eight different classes. The proposed models achieved an accuracy of 86.9% and 87.8% on the test set, respectively. Gupta et al. [30] introduced an approach aimed at automating the identification of GI tract diseases using DL. They leverage the Kvasirv2 for their investigation and employ EfficientNetB7 and ResNet50 techniques that have been pre-trained on ImageNet for feature extraction. In the categorization phase, they employ a Voting Classifier and report a peak accuracy of 88.19%. Furthermore, the authors contrast the outcomes of Wildwood and Random Forest algorithms on the Kvasir, demonstrating the efficacy of their proposed methodology. Yoshiok et al. [31] analyzed the performance of four different CNN methods (MobileNet V3, MobileNet V2, ResNet-50 and GoogleNet) in detecting esophagitis from endoscopic images in the Kvasir dataset. The study finds that GoogLeNet achieved the highest F1-score, while MobileNet V3 estimated esophagitis more rightly than the other methods based on the average true positive rate. The accuracy values obtained for the models were 84.6% for GoogLeNet, 84.2% for MobileNet V3, 83.3% for ResNet-50, and 83% for MobileNet V2. Agrawal et al. [32] suggested a architecture consisting of VGG and InceptionV3 for the classification of GI system abnormalities with endoscopic images. Following the experimental studies on the Kvasir to test the suggested method, an F1-score value of 84.7% was obtained. Gammulle et al. [33] improved a architecture for automated endoscopy image classification based on the ResNet-50. F1-score value of 89.7% was found with the Kvasir dataset used for the analysis of the classification accuracy of the architecture.

In addition to CNN-based methods, vision transformer (ViT)-based methods have been used in recent years. The ViT has brought about a significant transformation in the realm of DL. It employs attention mechanisms to enhance interpretability and efficiency across diverse domains, such as computer vision and natural language processing, marking a notable shift in the field. Some of the studies using these methods are as follows. Huo et al. [34] suggested a new DL network for medical image classification that combines the strengths of both self-attention-based Transformers and CNNs. The suggested method employs a hierarchical multi-scale feature fusion network known as HiFuse, comprising three branches. This network proves proficient in extracting global and local features across diverse semantic scales. HiFuse further integrates an adaptive hierarchical feature fusion block (referred to as the HFF block) to thoroughly merge semantic details across distinct scale features within each branch. The HiFuse Tiny, HiFuse Small, and HiFuse Base models attained accuracy rates of 84.85%, 85.00%, and 84.35%, respectively. Bai et al. [35] improved a ViT-based architecture for the classification of wireless capsule endoscopy images. They obtained 79.15% accuracy with the Kvasir-Capsule dataset utilized to evaluate the performance of the ViT-based architecture. Su et al. [36] proposed an image ViT-based feature pyramid network for polyp segmentation on endoscopy images. The performance of the ViT-based feature pyramid network was tested with the Kvasir dataset and an average Dice coefficient of 92.4% was obtained. Hosain et al. [37] used ViT to classify gastrointestinal diseases from curated colon images with wireless capsule endoscopy. They obtained an F1 score of 88.75% in experimental studies on a four-class dataset with Esophagitis, Polyps, Ulcerative colitis and healthy patients. Cao et al. [38] introduced the Sparse Attention Bidirectional Transformer as a model designed to identify GI diseases. Utilizing ViT, this model integrates sparse attention mechanisms to address the intricate nature of diverse GI diseases. Through experiments conducted on the HyperKvasir dataset, they observed an accuracy of 71.95% and an F1 score value of 63.38%.

## Materials and methods

### Kvasir dataset

**Fig. 1:**
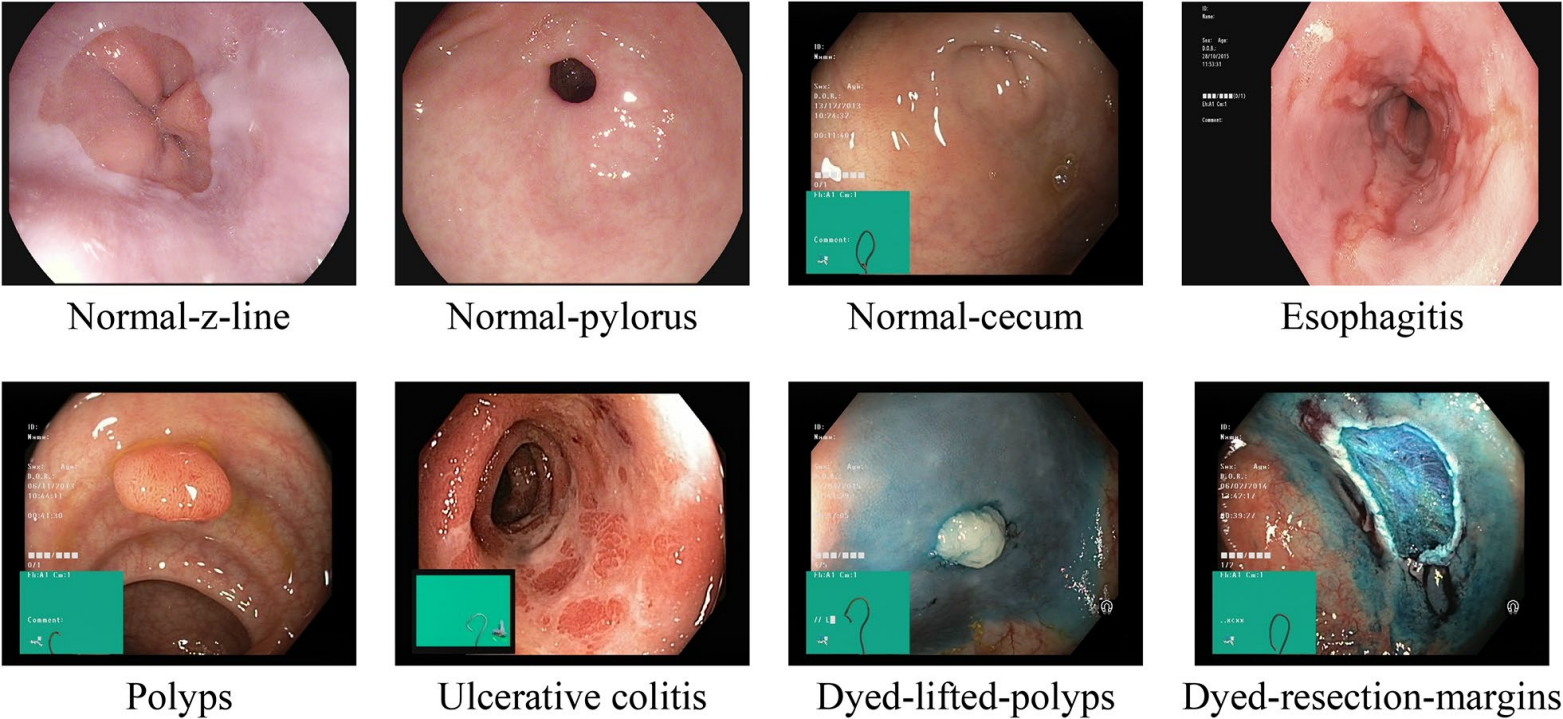
Sample images of classes in the Kvasir dataset [39]

The Kvasir dataset [39] is a collection of high-quality GI endoscopy images, designed to enable research on computer-aided diagnosis and automated disease detection in the GI tract. The dataset was gathered from endoscopic equipment used in hospitals under the Vestre Viken Health Trust in Norway, which serves a population of 470.000 people. The images were annotated by medical experts from the Cancer Registry of Norway (CRN), an independent institution under Oslo University Hospital Trust, responsible for cancer screening programs to prevent cancer deaths by detecting pre-cancerous lesions or cancers as early as possible. It includes images of several different gastrointestinal diseases, including polyps, ulcers, and inflammation, as well as normal tissue. The dataset consists of over 8000 images with annotations for various lesions and diseases. The Kvasir has been widely recognized as a valuable resource for advancing research in the field of GI endoscopy. One of the strengths of the Kvasir is the high quality of the images. The images were acquired using high-definition endoscopes, which provide high-resolution images with fine details. The Kvasir is publicly available and has been used in several benchmarking studies, enabling direct comparison of different methods and models. The dataset consists of eight classes, including Ulcerative colitis, Dyed-lifted-polyps, Normal (cecum, z-line, pylorus), Esophagitis, Dyed-resection-margins and Polyps. Sample images for each class in the Kvasir dataset are given in Fig. 1.

### Data preprocessing

Data augmentation (DA) is a commonly used method in Computer vision (CV) that involves applying transformations to the original images to create new images. These transformations can include rotations, translations, scaling, flipping, and other operations that can simulate real-world variations in the images. The primary objective of DA lies in expanding the size of the training dataset, thereby enhancing the performance of DL methods trained on these datasets. DA holds significant importance in CV as it plays a pivotal role in mitigating overfitting and enhancing the generalization capabilities of DL methods. Overfitting arises when a model becomes excessively attuned to the training data, rendering it incapable of generalizing to fresh, unseen data. Through the application of DA, we can generate new images that preserve the essential patterns and characteristics of the original images but introduce variations that facilitate the acquisition of more robust features while mitigating overfitting. Furthermore, data augmentation proves invaluable in addressing class imbalance issues that frequently plague numerous image datasets. By generating additional images for underrepresented categories, we can rectify the dataset’s imbalance and elevate the overall effectiveness of the method. Additionally, DA can optimize the training process by diminishing the necessity for gathering new data, a task that can be both costly and time-intensive.

Before training our model, we preprocessed the Kvasir dataset by merging the three normal classes, namely normal (cecum, pylorus and z-line) and into one class called “normal.” This decision was made to simplify the classification task by removing the need to distinguish between different parts of the gastrointestinal tract. However, this merging of classes resulted in a class imbalance problem, where the “normal” class had three times more images than the other classes. To address this class imbalance problem, we implemented two DA methods to five of the classes: polyps, esophagitis, dyed-resection-margins, dyed-lifted-polyps and ulcerative-colitis. Specifically, we mirrored and rotated these images by 180 degrees to create new images that could be used to balance the dataset. However, we did not apply these techniques to the “normal” class because it already had three times more images than the other classes due to the merging of the three normal classes. Additionally, we applied the random brightness to all six classes before the training process. One potential disadvantage of using the Kvasir dataset for training a model is the presence of text on the images. This issue may lead to increased difficulty in accurately classifying images and may hinder the performance of the model. Thus, it is essential to acknowledge this limitation and consider strategies to minimize the effects of non-relevant text on the model’s performance during training.

### Proposed spatial-attention ConvMixer (SAC) model

**Fig. 2:**
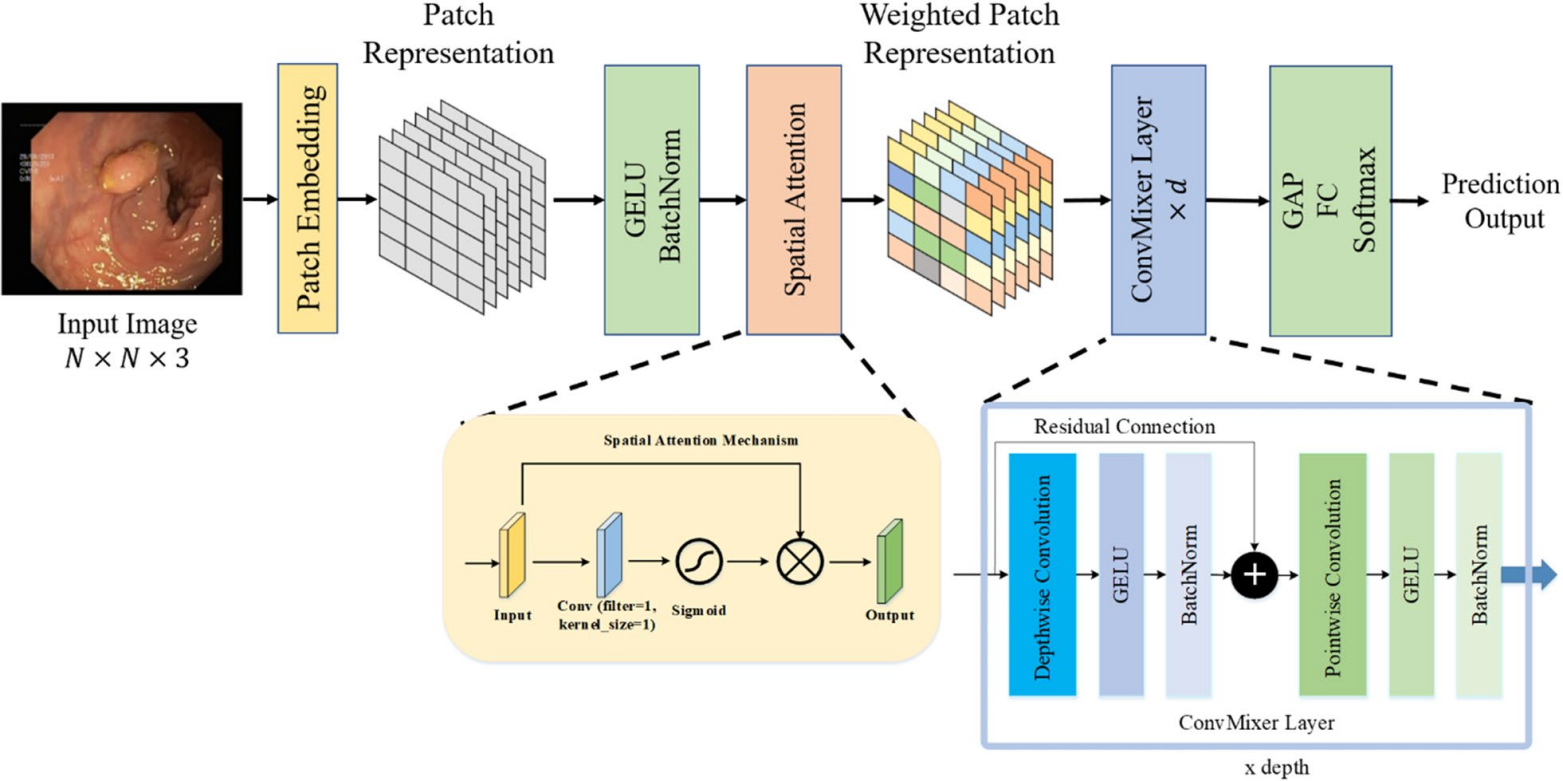
Proposed SAC model

Trockman et al. [40] argued that the source of high success in models such as ViT, MLPMixer, and Swin Transformer may be processing images by patching them. In this direction, with the proposed ConvMixer architecture, the input image is patched and powerful features that can achieve high performance are obtained. In this study, we propose a novel ConvMixer architecture that includes the spatial attention mechanism (SAM), which we call Spatial-Attention ConvMixer (SAC) to obtain stronger patches. The primary motivation behind SAC is to selectively focus on the most informative regions of the feature maps, enabling the network to identify the salient features more efficiently, leading to improved classification performance. The SAC model is given in Fig. 2. As seen in Fig. 2, patches were obtained from the image by patch embedding in the first stage of the SAC model. Patch Embedding is performed with a traditional convolution operation, as in the original ConvMixer. As a result of this process, patch representation data of size $\frac{N}{p} \times \frac{N}{p}\times h$ is obtained. Between these patch representations there are significant points, while at some points partially unimportant regions such as the frame edges of the image are represented. A selective approach to reveal important regions can enable ConvMixer layers to reveal more effective features. From this perspective, patch representations are passed to the Spatial Attention (SA) module. Basically, the SA module weights patch representations with pointwise convolution and sigmoid. As shown in "Spatial Attention Mechanism (SAM)" section, the sigmoid output is a weight matrix ranging from 0 to 1. Element-wise multiplication is performed between these weight matrix and patch representations. This process suppresses some insignificant details, while enhancing points that may be important. In this way, unlike the ConvMixer architecture, the SAC model strengthens the patch representations that may be important between patches. In the second stage of the proposed SAC model, the patch representations that pass through the SA module and are weighted are transferred to the ConvMixer layers. Here, pointwise convolution and depthwise convolution are applied, respectively. At the end of each convolution process, Gaussian Error Linear Unit (GELU) and Batch Normalization (BatchNorm) layers are used. The layer details of the SAC architecture are shown in Table 1. As seen in Fig. 2; Table 1, in the last part of the SAC model, a $64 \times 64 \times 256$ feature map obtained in ConvMixer layers is obtained. First of all, the global average pooling (GAP) layer was implemented to this feature map. Then, the classification prediction map was obtained with the fully connected layer and the softmax layer. As given in Table 1, the hyperparameters of the ConvMixer layer in the SAC model are 5 for the kernel size of the depthwise separable convolution and 256 for the filter number of the pointwise convolution. Finally, the ConvMixer layer has a depth of d = 8.

**Table 1:** Summary of the SAC model

Levels	Layers	Stride	Filter	Kernel size	Output
Input	Input layer	–	–	–	128, 128, 3
Patch embedding Patch size: 2	Conv2D	2	256	2	64, 64, 256
Spatial attention	PointWise Conv2D	1	1	1	64, 64, 1
	Multiply	–	–	–	64, 64, 256
ConvMixer layer Depth: 8	DepthWise Conv2D	1	–	5	64, 64, 256
	GELU	–	–	–	64, 64, 256
	BatchNorm	–	–	–	64, 64, 256
	Add	–	–	–	64, 64, 256
	GELU	–	–	–	64, 64, 256
	BatchNorm	–	–	–	64, 64, 256
	PointWise Conv2D	1	256	1	64, 64, 256
Classification block	GlobalAvgPool2D	–	–	–	256
	Dense	–	6	–	6

ConvMixer and SAM, which are involved in the design of the proposed SAC model, are discussed in detail in the subsections below.

#### ConvMixer

The ConvMixer (CM) is a simple convolutional architecture proposed as an alternative to the patch-based representation of Vision Transformers (ViT). The ViT achieves high performance through self-attention layers, but they have a quadratic runtime and require the use of patch embeddings. In contrast, the CM operates directly on patches as input and uses only standard convolutions for mixing steps. The CM protects resolution and equal size throughout the network and separates the mixing of channel and spatial dimensions [40].

The CM architecture outperforms both classical vision models such as ResNets and some corresponding MLP-Mixer and ViT variants, even with additions intended to make those architectures more performant on smaller datasets. The method is based on the idea of mixing, where depthwise convolution (DC) is used to mix spatial locations and pointwise convolution (PC) to mix channel locations. The method is instantiated with four hyperparameters: the hidden dimension, depth, kernel size, and patch size. The architecture is named after its hidden dimension and depth, like $CM-h/d$. The CM supports variable-sized inputs and is based on the idea of mixing, which is used in other architectures. These results suggest that patch embeddings themselves may be a critical component of newer architectures like ViT.

**Fig. 3:**
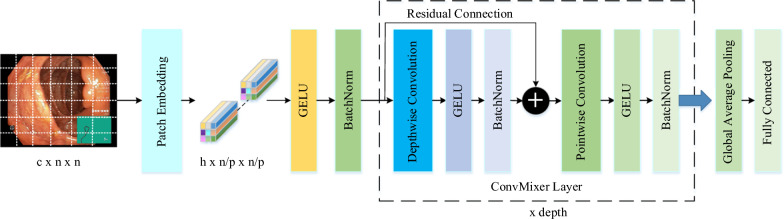
ConvMixer architecture

The CM architecture consists of three stages as shown in Fig. 3. The first stage of this architecture consists of a patch embedding layer and repeated applications of a fully-convolutional block. The patch embeddings are applied as convolution with input channels, kernel size, stride and output channels. The Patch embedding transforms an $n \times n$ image into a feature map of size $h \times n/p \times n/p$, where $p \times p$ is the size of the patch and $h$ is the number of filters used in the convolution layer [41]. Following the patch embedding layer, there’s the application of the GELU, succeeded by BatchNorm layers. The GELU activation function, like RELU, weights the inputs by magnitude rather than classifying them by their sign. It is a high-performance activation function. The second stage of the architecture is the CM block. This block is repeated for a predetermined number of depth times. The CM block consists of DC followed by PC, and each convolution is followed by an activation and post-activation BatchNorm. In this block, the DC is contained within the residual block. A residual block constitutes a structural unit wherein the outcome of the prior layer is combined with the output of a subsequent layer. The DC used within the CM block filters each input channel independently. It is used to mix the spatial dimensions of the image. PC is a convolution operation that allows filtering using $1 \times 1$ convolution to iterate over every single point or pixel in the image. It is used to mix information across the patches. Following numerous uses of this block, the GAP operation is executed to obtain a feature vector, subsequently fed into a softmax classifier. This is the third stage of the CM architecture [40].

#### Spatial attention mechanism (SAM)

**Fig. 4:**
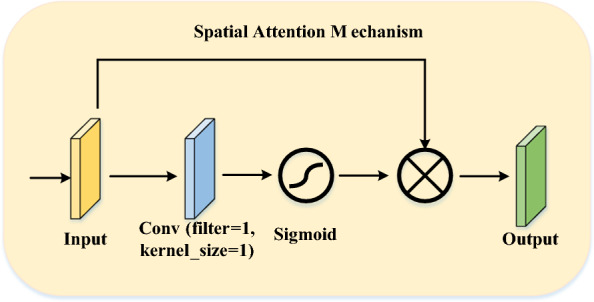
Spatial attention mechanism

Spatial attention is a mechanism that has been commonly used in recent years to increase the performance of CNN architectures in various CV tasks [42]. It allows the architecture to selectively focus on specific regions of the input image by assigning higher weights to relevant features while downplaying the importance of irrelevant ones. The primary function of spatial attention is to capture the interdependencies between different regions of an image by emphasizing the important regions and suppressing the less relevant ones. This is achieved through the use of a gating mechanism that generates a spatial map, which is multiplied by the input features to amplify or attenuate them [43]. This gating mechanism is typically implemented using a learnable parameter that is trained alongside the rest of the model. Spatial attention can be incorporated into different CNN methods, including CNNs and transformers. In CNNs, spatial attention can be added as a separate module after the convolutional layers, while in transformers, it is typically included as part of the self-attention mechanism. The SAM is shown in Fig. 4. In this mechanism, a convolution layer with a kernel size of $1 \times 1$ and a filter and sigmoid function are used to produce the final weights for each region on the feature map. Thanks to the $1 \times 1$ convolution, the depth dimension of each point in the feature map is gathered at one point. Then, matrix weights are obtained by applying the sigmoid function to this convolution output. Finally, the output feature map was acquired by implementing the element-wise multiplication of the obtained weights with the input.

## Results and discussion

Numerous experimental analyses have been conducted to thoroughly examine the performance of the SAC model. These empirical investigations are presented in this section. Following this, the section elaborates on hyperparameter settings. Subsequently, the SAC model is contrasted with studies in the Kvasir dataset literature and state-of-the-art models like ViT, Swin Transformer, ConvMixer, and MLPMixer. Finally, the SAC model behavior analysis was performed for the Kvasir dataset using GradCAM (Gradient-weighted Class Activation Mapping).

### Settings of hyperparameters

We utilize a specific arrangement of hyperparameters to train our SAC architecture using the TensorFlow library within the Google Colab, which includes a Tesla T4 GPU and 2× Intel(R) Xeon(R) CPU @ 2.30 GHz paired with 12GB of RAM, offering ample computational capability for our training requirements. Overall, we anticipate that the conjunction of hyperparameters, callbacks and optimizers with TensorFlow in the Google Colab setting will facilitate achieving cutting-edge outcomes with our SAC architecture. Our hyperparameters encompass validation split, image size, batch size, learning rate (lr), weight decay, number of epochs, filters, depth, kernel size, and patch size. Specifically, the proposed SAC model is divided into 70% training, 15% testing, and 15% validation dataset. The images were trained with an image size of $128 \times128$ pixels and a batch size of 32. Additionally, we set the learning rate (lr) to 0.001, the weight decay to 0.0001, and performed training for 25 epochs. The architecture incorporates 256 filters, a depth of 8 with a patch size of 2 and a kernel size of 5. Additionally, To optimize the architecture and minimize the loss function, we utilize the AdamW [44] optimizer. Aside from the hyperparameters, we incorporate two distinct callbacks to enhance the training procedure. The initial callback, ReduceLROnPlateau, functions to diminish the lr when the validation loss plateaus, preventing overfitting and ensuring stability in training. The subsequent callback, ModelCheckpoint, saves the model weights periodically throughout training, enabling us to preserve the best model according to validation accuracy, ensuring its availability for subsequent use.

The proposed SAC model was evaluated on the Kvasir dataset. The assessment of the SAC model’s efficiency relied on evaluation metrics like F1-score (F1s), precision (Pr), recall (Re) and accuracy (Acc). These metrics offer an objective quantitative measure, crucial in appraising a architecture’s predictive efficacy and identifying potential enhancement areas. Each criterion offers a specific viewpoint on the architecture’s performance, each with its particular strengths and drawbacks. Below, a comprehensive elucidation of these metrics is provided.

The metric of Acc (Eq. 1) serves as a fundamental evaluation measure, determining the proportion of correct predictions derived from the architecture. It is computed by dividing the count of accurate predictions by the overall number of predictions. Nonetheless, when dealing with imbalanced datasets, where the sample sizes in each class differ significantly, Acc can be misleading. The Pr (Eq. 2), a metric assessing the ratio of true positives (TP) among all positive predictions generated by the architecture, is calculated by dividing TP by the sum of false positives (FP) and TP. The Pr is particularly valuable in situations where the cost of an FP is significant. For example, in medical diagnosis, an FP can cause unnecessary tests and treatments, leading to additional expenses and discomfort for the patient. The Re (Eq. 3), a metric determining the ratio of TP within all the genuine positive samples in the dataset, is computed by dividing TP by the total of false negatives (FN) and TP. The Re is particularly useful when the cost of an FN is high. For instance, in disease diagnosis, an FN can lead to a delay in treatment, resulting in more severe symptoms or even death. The F1s (Eq. 4), a measure combining the Pr and Re through a harmonic mean, serves as a crucial metric to balance these factors, especially when dealing with imbalanced classes. This score offers a unified measure capturing both the Pr and Re, making it a powerful assessment metric for evaluating overall model performance.

1 $$Acc= \frac{TN+TP}{TN+FN+TP+FP},$$

2 $$Pr= \frac{TP}{TP+FP},$$

3 $$Re= \frac{TP}{TP+FN},$$

4 $$F1s=2 \times\frac{Pr \times Re}{Pr+Re}.$$

**Fig. 5:**
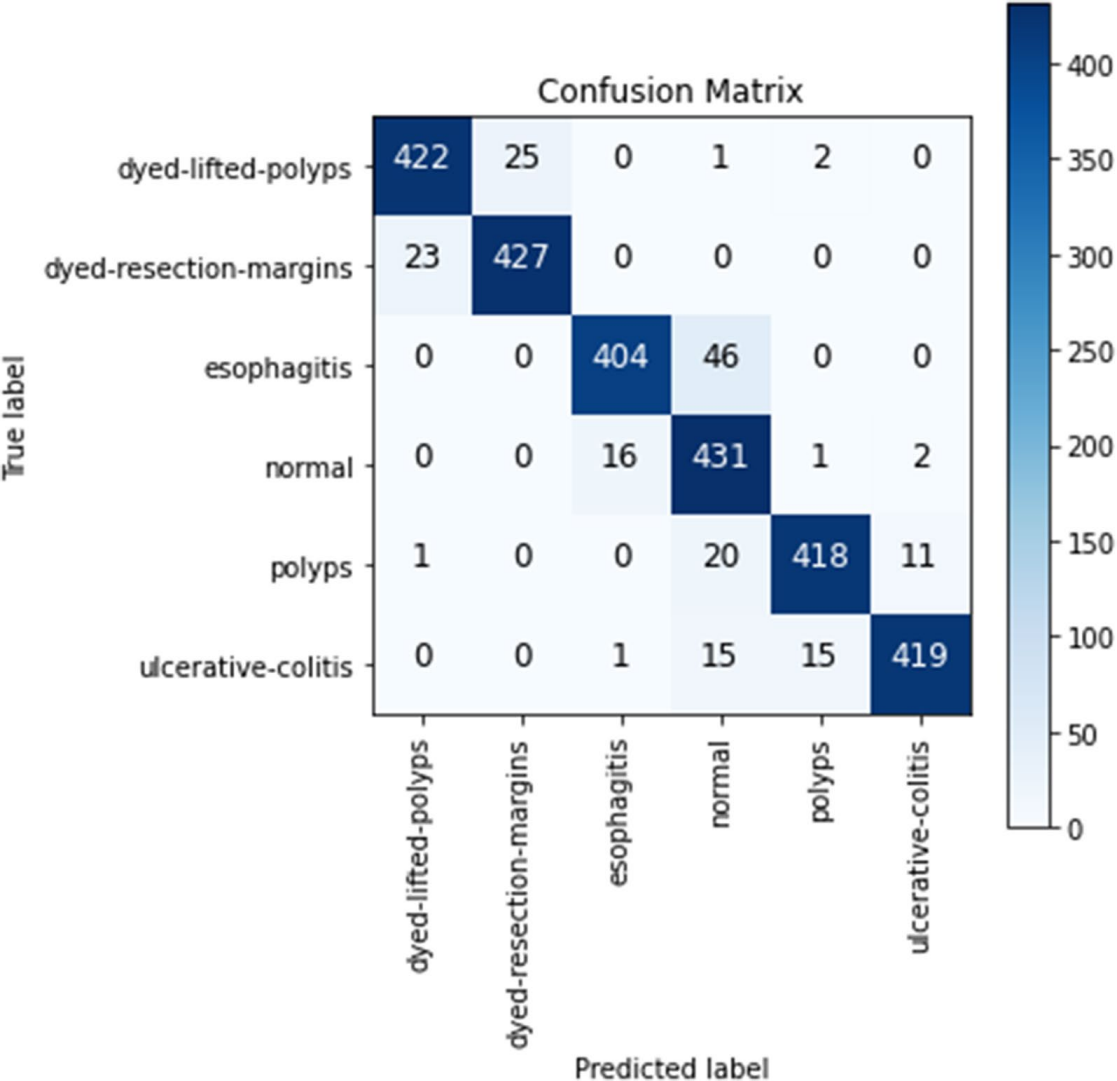
The multi-class confusion matrix of the proposed SAC model

FN, FP, TP and TP values are obtained from the confusion matrix. The confusion matrix of the SAC architecture is given in Fig. 5. Considering the confusion matrix in Fig. 5, it shows that 422 images from 450 Dyed-lifted-polyps images were predicted correctly. Similarly, it appears that 427 from 450 Dyed-resection-margins images, 404 from 450 Esophagitis images, 431 from 450 Normal images, 418 from 450 Polyps images, and 419 images from 450 Ulcerative colitis images appear to be predicted correctly. In addition, the Pr, Re, and F1s values for each class with the proposed SAC model according to the confusion matrix are given in Table 2.

**Table 2:** Class-based classification report for the proposed SAC model

Classes	Pr (%)	Re (%)	F1s (%)	Support
Dyed-lifted-polyps	94.62	93.78	94.20	450
Dyed-resection-margins	94.47	94.89	94.68	450
Esophagitis	95.96	89.78	92.77	450
Normal	84.02	95.78	89.52	450
Polyps	95.87	92.89	94.36	450
Ulcerative colitis	96.99	93.11	95.01	450
Overall Acc			93.37	2700
Macro Avg	93.66	93.37	93.42	2700
Weighted Avg	93.66	93.37	93.42	2700

### Experimental results

In this section, the SAC model is compared with state-of-the-art methods such as ConvMixer [40], Vanilla ViT (VVT) [45], Swin Transformer [46], MLPMixer [47], ResNet50 [48] and SqueezeNet [49]. Then, considering the recent studies for the Kvasir dataset, the SAC model was analyzed. Then, the latest studies for the Kvasir dataset and the SAC model were compared.

Table 3 presents the performance comparison of several state-of-the-art DL architectures on the Kvasir dataset in terms of Re, Pr, Acc and F1s. The objective is to classify these images into their respective classes using DL architectures. VVT achieved an Acc of 79.52%, Re of 80.0%, Pr of 80.0%, and F1s of 80.0%. VVT is a popular transformer-based model that has shown perfect performance in CV tasks. However, compared to other models such as ConvMixer and SAC, VVT has a lower accuracy on this dataset. One possible reason is that the Kvasir dataset is highly complex and diverse, and VVT might not be able to capture all the relevant features effectively. The second model is the Swin Transformer, which achieved an Acc of 74.52%, Re of 75.0%, Pr of 75.0%, and F1s of 74.0%. Swin Transformer is a recently proposed model that aims to address the limitations of the standard transformer architecture, such as high memory requirements and limited receptive fields. Despite its promising results on other datasets, Swin Transformer underperformed on the Kvasir dataset. This might be due to the fact that the Kvasir dataset has unique characteristics that require more specialized models. ConvMixer achieved F1s of 92.0%, Acc of 92.48%, Pr of 93.0%, and Re of 92.0%. ConvMixer is a novel architecture that replaces the self-attention mechanism in transformers with convolutional layers. This allows the model to learn local features efficiently and capture spatial dependencies. As shown in the table, ConvMixer outperformed most of the other models, including VVT and Swin Transformer, on the Kvasir dataset. This suggests that ConvMixer is well-suited for complex medical image classification tasks. The fourth model is MLPMixer, which achieved an Acc of 63.04%, Re of 63.0%, Pr of 67.0%, and F1s of 63.0%. MLPMixer is another novel architecture that replaces the self-attention mechanism in transformers with MLPs (multi-layer perceptrons). MLPs are widely used in traditional neural networks and are known for their ability to learn complex functions. However, MLPMixer did not perform well on the Kvasir dataset, suggesting that the self-attention mechanism might be better suited for this task. The fifth model is ResNet50, which achieved an Acc of 87.44%, Re of 87.0%, Pr of 88.0%, and F1s of 87.0%. ResNet50 is a popular CNN that has been shown to be effective in many CV tasks. However, on the Kvasir dataset, ResNet50 was outperformed by ConvMixer and SAC. This might be due to the fact that ResNet50 is a relatively older model and might not be optimized for the unique characteristics of the Kvasir. SqueezeNet acquired an Acc of 85.59%, Re of 86.0%, Pr of 86.0%, and F1s of 86.0%. SqueezeNet is another CNN that aims to reduce the memory and computational requirements of DL methods. While SqueezeNet achieved good performance on the Kvasir dataset. The proposed SAC model acquired the highest Acc of 93.37%, Re of 93.37%, Pr of 93.66%, and F1s of 93.42% on the Kvasir dataset among all the methods compared in the table.

The proposed SAC model is a novel model that combines the strengths of two different types of DL models, namely spatial attention and ConvMixer. The spatial attention is a mechanism that enables the method to selectively concentrate on particular regions of the image while ignoring irrelevant regions. This is achieved by assigning different weights to different regions of the image, based on their relevance to the task at hand. In the proposed SAC model, spatial attention is implemented to the input of the ConvMixer layers, allowing the method to focus on the most relevant features in the input images. The ConvMixer, on the other hand, is a recently proposed architecture that replaces the self-attention mechanism in transformers with convolutional layers. The ConvMixer is well-suited for image classification tasks, as it allows the model to learn local features efficiently and capture spatial dependencies. In the proposed SAC model, ConvMixer layers are used as the main building blocks of the model, which allows it to extract relevant features from the input images. The combination of the spatial attention mechanism and ConvMixer in the proposed SAC model allows the model to effectively learn both local and global features from the input images. This is particularly important for medical image classification tasks, as the relevant features might be distributed across different regions of the image. When the classification accuracies of the proposed SAC model and the ConvMixer architecture are compared, it is seen that the SAC model achieves 0.89% better accuracy. This increase in accuracy is due to the spatial attention mechanism added to the proposed model. With this result, it is clear that the spatial attention mechanism improves the performance of the SAC model by enabling it to focus on the most relevant regions of the image.

In addition, the number of trainable parameters for all models is given in Table 3. The proposed SAC model has 593.415 parameters, while ConvMixer has 593.158 parameters. The spatial attention mechanism in the proposed SAC model increased the number of trainable parameters by 257 and contributed 0.89% to the classification accuracy. On the other hand, the model with the lowest parameters is Swin transformer. However, the Swin transformer model obtained lower classification results than both the proposed SAC model and ConvMixer. Among the other models, the highest trainable parameter was found with ResNet50 with 49 million.

**Table 3:** The performance comparison of several state-of-the-art DL models on the Kvasir dataset

Model	Acc (%)	F1s (%)	Pr (%)	Re (%)	Parameters
Vanilla ViT	79.52	80.0	80.0	80.0	769,222
Swin Transformer	74.52	74.0	75.0	75.0	396.630
ConvMixer	92.48	92.0	93.0	92.0	593.158
MLPMixer	63.04	63.0	67.0	63.0	1.633.030
ResNet50	87.44	87.0	88.0	87.0	49.226.502
SqueezeNet	85.59	86.0	86.0	86.0	1.254.430
**SAC (Ours)**	**93.37**	**93.42**	**93.66**	**93.37**	593.415

Bold indicates best result

A comparison of the classification accuracies obtained by various methods using the Kvasir dataset is presented in Table 4. The comparison between the SAC model and the existing methods was performed based on common evaluation metrics and identical data. As demonstrated in Table 4, the SAC architecture yielded a classification Acc of 93.37%, outperforming the other methods. Of the other approaches evaluated, the method yielding the closest performance to the SAC model was reported by Lonseko et al. [26] with a classification Acc of 93.19%. The SAC model surpassed this performance with a margin of 0.18%. The least successful approach in terms of classification accuracy was FocalConvNet, developed by Srivastava et al. [24], which achieved a classification Acc of 63.73%. When other studies using the Kvasir dataset are examined, the studies with a classification Acc of less than 90% are as follows: Sandler et al. [50] 79.15%, Pozdeev et al. [51] 88%, Agrawal et al. [52] 83.8% and Zhang et al. [53] 88.6%. The studies that have been obtained by using Kvasir data and with a classification result of more than 90% are as follows: Lonseko et al. [26] 93.19%, Fonolla et al. [54] 90.20%, Liu et al. [55] 93%, Wang et al. [56] 92.81% and Zhang et al. [57] 90.4%. When all the methods used for comparison in the literature are examined, the proposed SAC model shows higher classification performance than other methods.

**Table 4:** Comparison results with studies using the Kvasir dataset in the literature

Author	Methods	Dataset	Acc (%)
Srivastava et al. [24]	FocalConvNet	Kvasir	63.73
Lonseko et al. [26]	Deep CNN based SAM	Kvasir	93.19
Sandler et al. [50]	MobileNetV2	Kvasir	79.15
Pozdeev et al. [51]	Custom CNN for two-stage classification	Kvasir	88.00
Agrawal et al. [52]	Combined VGG, ResNet50, InceptionV3, Xception, MobileNet	Kvasir	83.8
Zhang et al. [53]	Regression-based CNN	Kvasir	88.6
Fonolla et al. [54]	Multi-model classification	Kvasir	90.20
Liu et al. [55]	Transfer learning framework	Kvasir	93.00
Wang et al. [56]	Efficient channel attention (ECA) module	Kvasir	92.81
Zhang et al. [57]	Single shot MultiBox Detector for gastric polyps network	Kvasir	90.4
Gjestang et al. [58]	Teacher–student framework	HyperKvasir	89.3
Gjestang et al. [58]	Teacher–student framework	Kvasir Capsule	69.5
**SAC (Ours)**	**Spatial-attention ConvMixer**	**Kvasir**	**93.37**

Bold indicates best result

### GradCAM visualization of the proposed SAC model on the Kvasir Dataset

**Fig. 6:**
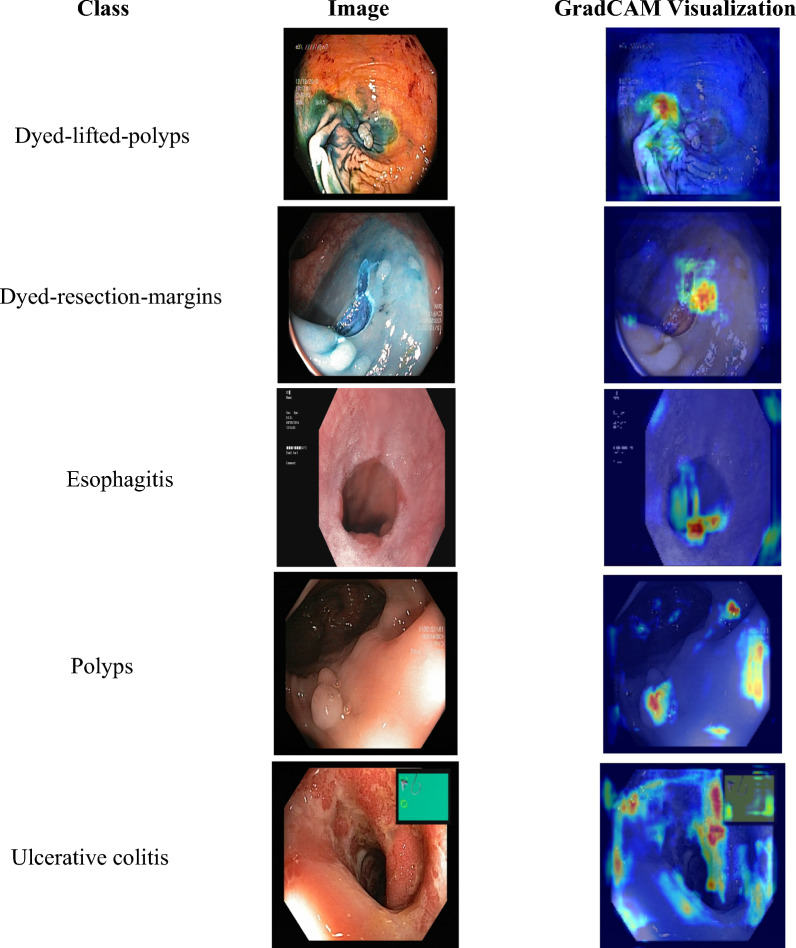
GradCam visualization for each class

In this experimental study, we present GradCAM (Gradient-weighted Class Activation Mapping) [59] visualizations for each class in the dataset to provide further insight into how the proposed SAC model makes its predictions. GradCAM is a visualization technique that provides insights into how a CNN makes its predictions by highlighting the regions of the input image that are most important for the network’s decision. To create a GradCAM representation, the gradient of the score for the target class is computed concerning the feature maps from the final convolutional layer. These gradients are then weighted by their importance to the output class, and the weighted gradients are summed to obtain the class activation map. This map is then overlaid onto the original input image to highlight the regions that are most important for the network’s decision for a particular class. GradCAM proves valuable in interpreting CNN architectures as it provides insights into the decision-making process, aiding in pinpointing any potential biases or shortcomings within the architecture [59]. In this context, we generated GradCAM visualizations for each class in the Kvasir dataset to gain insights into how the proposed architecture is making its predictions. These visualizations allowed us to identify the important regions of the image associated with each class and provided a more interpretable way of understanding the model’s behavior. The visualizations in Fig. 6 show that the SAC architecture is able to identify the relevant regions in the input image with high accuracy. The regions highlighted by the GradCAM technique correspond well with the anatomical structures and pathologies present in the images. This suggests that the proposed architecture is able to capture the salient features of the input images, which are critical for accurate classification. Moreover, the visualizations also reveal the robustness of the proposed architecture to variations in image quality and lighting conditions. The architecture is able to identify the relevant regions in the input images even when they are of low quality or have poor lighting. This demonstrates that the proposed architecture is capable of generalizing well to new, unseen images. The GradCAM visualizations presented provide valuable insights into the inner workings of the proposed architecture. They demonstrate that the model is capable of accurate and robust classification and that it is able to identify the relevant regions in the input images with high accuracy. These findings have important implications for the medical field, where accurate and reliable classification of medical images is critical for effective diagnosis and treatment [59, 60].

## Conclusions

This study aimed to improve and evaluate DL methods for automatic classification and detection of GI diseases using the Kvasir dataset. The dataset contains over 8000 high-quality GI endoscopy images, including several different GI diseases and normal tissue, and has been commonly used in recent years for developing and evaluating DL methods. The high quality of the images, acquired using high-definition endoscopes and including annotations for various lesions and diseases, is one of the strengths of the Kvasir dataset.

In order to improve the performance of DL methods on this dataset, several DA methods were employed, including random flipping, random rotation, and random brightness. Furthermore, to address the class imbalance problem resulting from the merging of three normal classes into one class, two DA methods were implemented to five of the classes. This helped to increase the diversity of the training data and improved the generalization performance of the methods.

The proposed model, called the Spatial-Attention ConvMixer (SAC), is a new DL method that incorporates both spatial attention and ConvMixer blocks. The SAM allows the network to selectively focus on the most informative regions of the input images by weighting the importance of each spatial location in the feature maps. This mechanism has been shown to be particularly effective for medical image analysis tasks, where the most informative regions of the images are often critical for accurate diagnosis and treatment planning. The ConvMixer blocks, on the other hand, provide a powerful feature extraction capability that allows the model to capture complex patterns and structures in the input images.

The SAC model achieved state-of-the-art results on the Kvasir, with an Acc of 93.37%, outperforming several other DL methods, including the Vanilla ViT, Swin Transformer, ConvMixer, MLPMixer, ResNet50, and SqueezeNet. The SAC architecture also achieved high F1s, Pr, and Re scores, indicating that it is capable of accurately detecting and classifying different gastrointestinal diseases.

The findings of this investigation establish the efficacy of the SAC model for automatic identification and classification of GI ailments using DL models on the Kvasir dataset. Moreover, the incorporation of DA methods, including random flipping, rotation, and brightness, can substantially enhance the performance of DL methods, particularly in scenarios with class imbalance. Additionally, attention mechanisms, such as the spatial attention mechanism proposed in this study, can aid in improving the interpretability and precision of DL methods for medical image analysis tasks.

The SAC model can have several potential applications in clinical practice, such as assisting medical professionals in diagnosis and treatment planning for different gastrointestinal diseases. Moreover, the SACarchitecture can be adapted and extended to other medical image analysis tasks, like the classification and detection of other types of cancers, and can potentially lead to the development of more accurate and reliable DL methods for medical image analysis.

## Data Availability

Data will be made available on request.
